# A Novel Case and Treatment

**Published:** 1870-04

**Authors:** S. J. Cobb

**Affiliations:** Nashville, Tenn.


### ARTICLE II.

### *A Novel Case and Treatment.*

By Dr. S. J. Cobb, Nashville, Tenn.

A lady, who was so unfortunate as to lose, at the age of twenty, all of her upper teeth except the three roots of the second left superior molar, over which she has worn a plate for ten or twelve years, called upon a dentist a short time ago for the purpose of having her plate refitted, and he very naturally suggested the necessity of removing these loose roots from the mouth, which she readily consented to, and in his efforts to remove them pushed them up into the antrum. The operation becoming a little painful to the patient and frightful to the operator, was no longer persisted in, but in about eight hours from that time they were blown out at the nose.

I presume the floor of the antrum covering these roots had been necrosed and partially exfoliated for two or three years, from the fact that she has since called upon me to operate and treat for diseased antrum. In diagnosing the case, I found from necrosis and exfoliation not only the floor of the antrum covering these roots destroyed, but a portion of the ethmoid and inferior turbinated bones, making an opening sufficiently large for these roots to pass into the nasal fossa, from which they passed out at the nose. I also found there had been a constant, copious fetid discharge through the nose for five years, following an attack of erysipelas of the face.

In operating I removed all of the necrosed and exfoliated bone, after which I passed well up into the parts a small piece of sponge thoroughly saturated in two parts carbolic acid and one of tinc. of iodine. I then diluted with soft water the acid and iodine solution, and syringed the parts well and sent my patient home, with directions to use as a wash for the parts the compound of acid, iodine and water, also to keep the parts well cleansed with tepid water, and take in the way of general treatment, ten grains of blue mass, followed by one or two doses of citrate of magnesia, after which to take three times a dose of twenty drops of syrup of the iodide of iron. For two or three days after the operation the discharge slightly increased as I anticipated, having used the strong solution of acid and iodine for the purpose of producing a sufficient sloughing to bring away such small detached pieces of bone as might remain somewhat attached to the soft parts. In the course of a week the discharge commenced decreasing rapidly, at which time I fitted a plate and teeth to the jaw, covering the part well for the purpose of keeping particles of food and other matter out of the antrum.

At the end of twenty days treatment the discharge ceased entirely, and upon examination the secretions were found to be as healthy as they ever were.

---

